# SQUIRREL: Balancing design automation and user interaction in a computational tool for designing segmented concrete shells

**DOI:** 10.1177/14780771251316128

**Published:** 2025-02-21

**Authors:** Eduardo Costa, Robin Oval, John Orr, Paul Shepherd

**Affiliations:** 1 School of Architecture and Environment, University of the West of England, Bristol, UK; 2 Department of Materials, Mechanics, Management & Design, 2860 TU Delft, Delft, The Netherlands; 3 Department of Engineering, 2152 University of Cambridge, Cambridge, UK; 4 Department of Architecture and Civil Engineering, 1555 University of Bath, Bath, UK

**Keywords:** Design automation, design space, design exploration, computational design, parametric modelling, construction-aware design, digital fabrication

## Abstract

The Automating Concrete Construction (ACORN) project explored digital workflows from the design to the construction of reinforced concrete building floor elements, reducing carbon emissions and increasing efficiency of building processes. The resulting digital tool, named SQUIRREL, enabled the design of shells, composed of prefabricated segments, through an interactive framework, composed of parametric design tools, and informed by architectural, structural, and construction requirements, including building integration, fabrication, transport, assembly, and resource reuse. This paper presents the design and implementation of the SQUIRREL tool, focusing on the main design tasks for a segmented reinforced concrete shell, including formfinding and segmentation layout definition. This paper also documents the development and application of a Design Space Visualisation module within SQUIRREL, which streamlined parametric studies used to inform the design decisions behind the modelling and implementation process. Finally, we discuss the right balance between design automation and user interaction, which should inform the development of future construction-aware computational design tools.

## Introduction

Computation is empowering the Architecture, Engineering and Construction (AEC) sector with tools to mitigate climate change. They enable the design of better performing buildings through efficient and accurate simulation.^1^ Digital fabrication technologies are currently being explored to increase productivity of construction processes, while reducing harmful emissions and consumption of non-renewable resources.^2^ The reduction of embodied carbon has become crucial to enable the construction industry achieving its net zero targets.^3^ Computational design techniques provide the increased accuracy, efficiency and productivity necessary for such reduction, by automating design processes and enhancing design exploration through algorithmic and parametric approaches. Such automation encompasses structural optimisation and the integration of construction constraints, which enables the design of more efficient building elements.^4^

### Design automation

In spite of the advantages of design automation, research suggests that too much automation can prevent designers from exploring valid design options. Consider optimisation as a paradigm of design automation. Black-box optimisation approaches, such as genetic algorithms and direct search, are beneficial when estimating the gradient is too time-consuming, requiring the evaluation of many design candidates.^5^ Even though these optimisation approaches produce faster results, they inherently hide potentially promising design solutions. In that regard, development of hybrid approaches, like performance maps for example, have shown that design space exploration and architectural design optimization can be improved by providing designers with an overview of the design space and corresponding fitness landscape, instead of presenting only a subset of design solutions, and allowing designers to switch back and forth between manual and automatic exploration of design spaces.^6^

This paper presents a computational design tool named SQUIRREL, as an example of a hybrid design automation process. The aim of SQUIRREL is to facilitate the design and manufacturing of concrete segmented shells in a fully digital workflow, informed by architectural, structural and construction requirements, including building integration, off-site fabrication, transport, assembly, and resource reuse. SQUIRREL is implemented as a plugin for visual programming environment Grasshopper,^7^ By combining methods like parametric modelling, simulation and optimisation, and exploring interoperability with other GH plugins. The resulting design automation process features both fully automated tasks as well as semi-automated tasks that benefit from user-interaction.^8^

While historically design automation systems have been developed as ‘black box’ solutions, where meaning, context, and design intent are hidden in design processes, the advent of visual programming solutions such as Grasshopper have been shown to improve transparency in design automation systems,^9^ which can be further enhanced by adopting a modular approach to the development of visual scripts.^10^ Within the use of Visual Programming, transparency and automation also vary according to the design methods used. Considering a definition of Level of Automation (LOA) for AEC-related design activities,^11^ SQUIRREL would be working in-between LOA level 3, corresponding to the integration of parametric design methods to generate multiple options, and LOA level 4, corresponding to the integration of simulation and optimisation techniques towards further narrowing down the design options.

### Research project

SQUIRREL was developed in the scope of a research project called Automating Concrete Construction (ACORN). Following the remit of the UK Government’s Construction 2025 strategy,^12^ the project explored digital workflows from the design to the construction of concrete building elements, to reduce emissions and increase efficiency.^13^

Concrete is the world’s most widely used human-made material, accounting for more than 7% of global CO_2_ emissions, due to a wide range of applications.^3^ In the UK, the construction industry is responsible for nearly half of the country’s carbon emissions.^14^ One way of reducing concrete-related carbon emissions is by reducing the volume of concrete over-used due to inefficient design and construction. In that respect, the project explored the potential for structurally efficient non-prismatic geometries to substantially reduce the amount of concrete in building elements. The project focused on segmented thin concrete shells as floors, leveraging computational design and digital fabrication methodologies to automate their production.

One of the project’s successes was the construction of a full-scale, 4.5 m x 4.5m demonstrator prototype of a segmented shell, named ‘OAK’, produced at the National Research Facility for Infrastructure Sensing (NRFIS) of the University of Cambridge, which illustrates how automating concrete construction can help drive the industry towards net-zero.^15^

### Contribution and outline

This paper focuses on the process of designing and implementing the SQUIRREL tool, including reflections on decisions made in the development process regarding design automation towards ensuring accuracy between the digital and physical, while facilitating user interaction. This deep-dive into the development of the SQUIRREL tool leads to a discussion about the right balance between design automation and user interaction, which should inform the development of similar computational design tools.

Section titled “Computational design framework” provides an overview of the tool’s computational implementation, focusing on the plugin’s architecture, its various components and respective usability. Section titled “Exploring shell shapes” focuses on how SQUIRREL is used to define the shape of segmented shells, whereas Section titled “Exploring segmentation layouts” addresses their segmentation, both using the design of the OAK prototype as a use case.^16^ Section titled “Conclusion” concludes the paper with a discussion of the benefits and caveats of automating optimisation processes in the design process.

## Computational design framework

**Figure 1.**
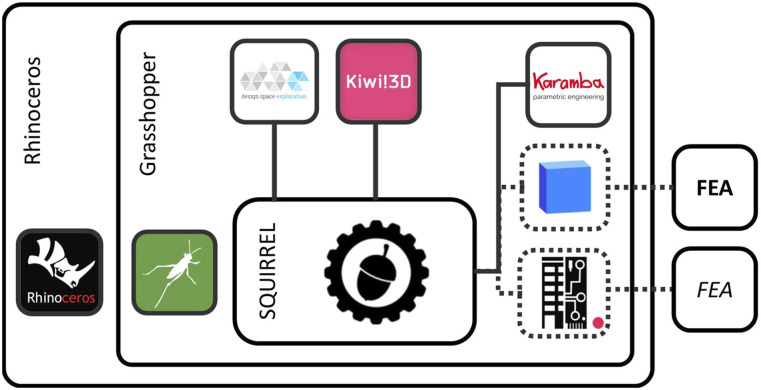
SQUIRREL plugin architecture.

SQUIRREL is a flexible tool composed of different modular components, integrated into the Grasshopper for Rhinoceros3D (v1.0.0007) ecosystem,^7^ which provides a parametric modelling framework to generate and explore efficient designs through digital processes.^17^ Implemented as a Grasshopper (GH) plugin, SQUIRREL makes use of three additional GH plugins, namely Kiwi!3D v0.5.0^18^ (Kiwi) for iso-geometric analysis (IGA), Karamba3D v1.3.3^19^ (Karamba) for structural Finite Element Analysis (FEA), and Design Space Exploration v1.4 (DSE), a toolbox for data-driven design that enables the generation and manipulation of design catalogs.^20^ Figure 1 illustrates SQUIRREL’s architecture as a plugin for Grasshopper, including its current dependencies from other plugins and potential alternatives (dashed outlines).

The SQUIRREL plugin is shared in a repository on GitHub (github.com/automated-construction),^21^ which provides version control and facilitates the plugin’s distribution. This repository hosts the plugin files as well as the source code, in the spirit of open source. Example files that illustrate how to use the plugin are also made available, as is common practice for GH plugin development.

### Dependencies

Both Karamba and Kiwi perform structural modelling and analysis in a parametric design workflow. However, their differences justify the use of both plugins, rather than selecting only one. On the one hand, Kiwi operates on NURBS geometry, which is Rhinoceros’ native geometry type, whereas FEA software such as Karamba typically requires a triangular mesh as an input for analysis.^22^ Maintaining the geometry as NURBS avoids possible inaccuracies derived from converting NURBS to mesh.^23^ It also allows for a wider range of subsequent design operations available in Rhinoceros and GH, since it is substantially more straightforward to convert NURBS to mesh when compared to the inverse conversion. On the other hand, Karamba provides a wider range of modelling inputs and analysis outputs than Kiwi, including spring elements and more detailed principal stress data, such as vector directions and values. Therefore, structural analysis tasks are performed using Karamba.

The use of existing plugins in the development of SQUIRREL, namely of Karamba and Kiwi, enabled a more immediate experimentation with different structural performance parameters and processes, rather than recreating some of its functionalities. However, the dependence on third-party Grasshopper plugins, often resulting from individual efforts in the scope of specific projects, can generate scalability issues, versioning issues, and vendor lock-ins.^24^ Also, even though the cost of a Karamba license is low relative to the cost of a licence of its parent software Rhino, the fact of being a paid licence might pose as an obstacle to the adoption of SQUIRREL. Therefore, in the spirit of open-source and its potential to create innovation and improve efficiency in the AEC sector,^24^ future iterations of SQUIRREL and its eventual transition to a finalised product should consider open-source alternatives to the licenced plugins currently in use. Such alternatives might include the integration of interoperability platforms such as BHoM or Speckle, both of which provide interfaces with a variety of FEA software^24,25^ (Figure 1, right-hand side).

Additionally, a custom tool was developed for Design Space Visualisation (DSV), which is implemented as an extension of the existing Design Space Exploration (DSE) plugin for Grasshopper,^20^ and integrated into SQUIRREL. DSV automates the generation of multidimensional design and objective spaces and generates two- and three-dimensional graphs from those spaces within Grasshopper, removing the need for additional external software typically used for this purpose, such as MATLAB, which would interrupt the continuous workflow needed for interactive design exploration.

### SQUIRREL components

**Figure 2.**
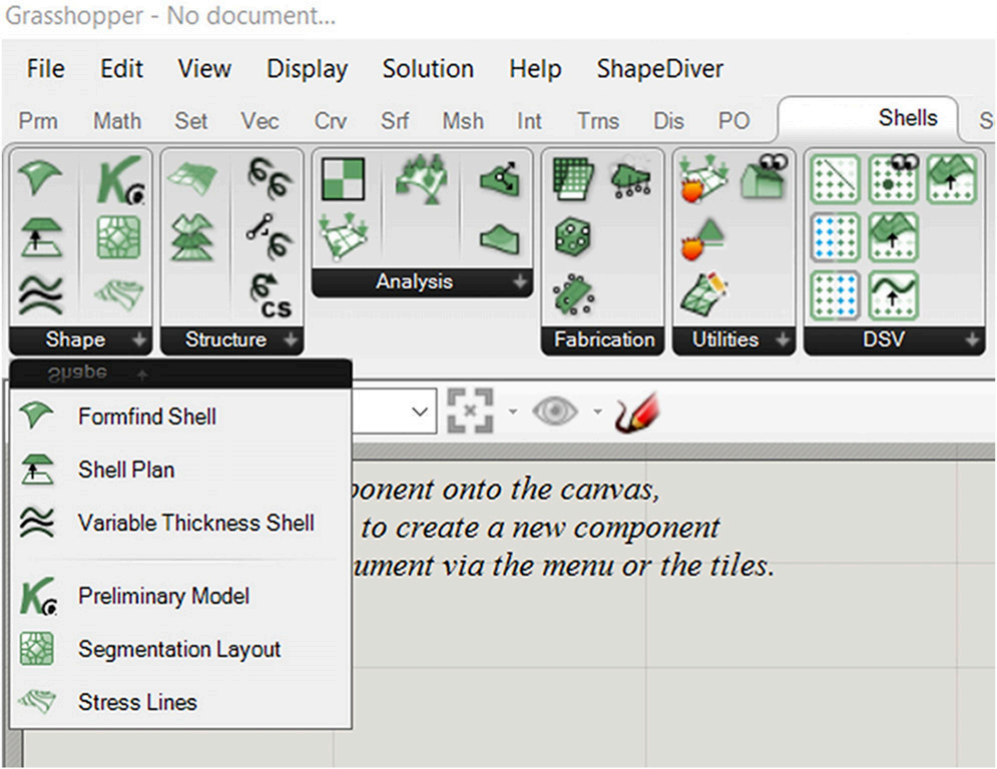
SQUIRREL toolbar.

The SQUIRREL plugin provides custom-built GH components that automate tasks necessary to design segmented shells, namely Shape modelling, Structure modelling, structural Analysis, and Fabrication processes simulation, sorted by their corresponding tabs (Figure 2). The Utilities tab includes components for model inspection and geometry visualisation, whereas the DSV tool offers visualisation of design spaces.

The plugin is written in C# and makes use of RhinoCommon and Grasshopper API for integration of geometrical operations, as well as K3D’s API for integration of FEA functionality. Kiwi!3D does not provide an API for accessing the plugin’s code library. Therefore, its functionality was accessed by using the NodeInCode namespace in RhinoCommon, which provides access to all Grasshopper runtime components, and allows developers to replicate their behaviour within a C# script.^26^ Therefore, while the use of SQUIRREL still requires having Karamba and Kiwi installed, designers can create design workflows by combining only SQUIRREL components and native GH components. Additionally, the components responsible for DSV functionality were implemented on a modified DSE library, waiving the need to install the DSE plugin.

While developing SQUIRREL, there has been an effort to fully automate some of the more repetitive tasks. These include calculations for structural analysis through Karamba, which already encapsulates most of the functionality needed to perform FEA. SQUIRREL furthers the encapsulation effort by automating the assembly of Karamba structural models, relying on the fact that segmented shells are a specific type of structural model, and therefore repeatable and prone to automation. In this case, automation is particularly beneficial considering the project’s aim to enable mass design and production of more sustainable building floors with a variety of layouts, rather than a single landmark concrete shell. Similarly, SQUIRREL encapsulates the Kiwi modelling process necessary to form-find a single component, which would otherwise be composed of dozens of Kiwi and GH components. Such encapsulation simplifies user interaction by greatly reducing the number of inputs, since most of which can be extracted from the input geometry.

**Figure 3.**
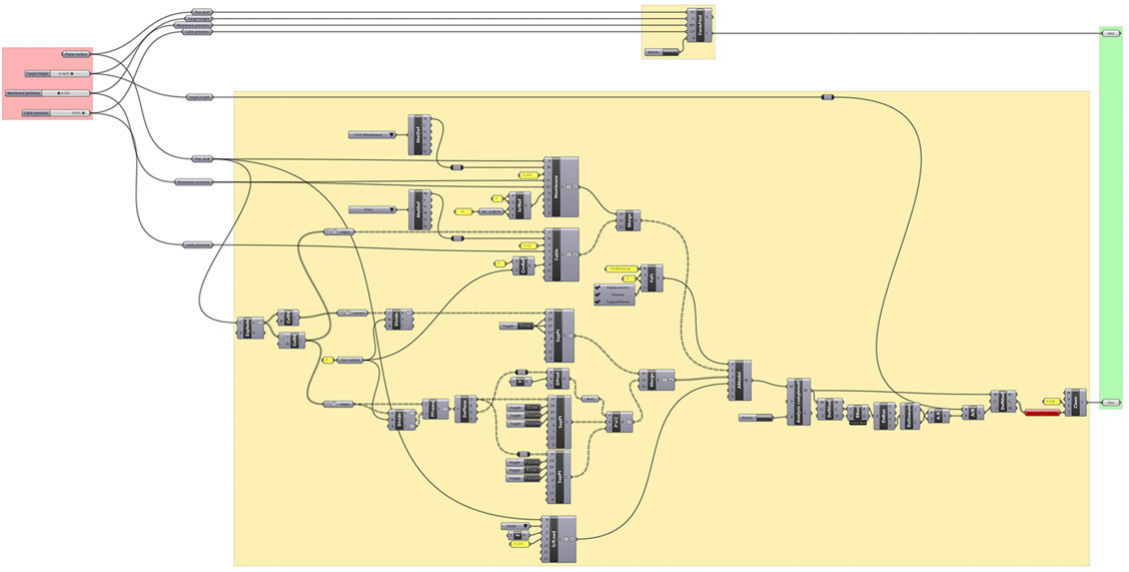
Squirrel’s Formfinding component and equivalent setup (in yellow) using Kiwi and Grasshopper components.

The “Formfind Shell” component is a good example of automation in assembling the structural model needed for the formfinding process. Figure 3 illustrates the comparison between the Formfinding component and equivalent setup consisting of more than 40 Kiwi and Grasshopper components.

### Hierarchy of the design process

SQUIRREL implements a hierarchical representation of design processes that correspond to different Scenarios (such as operational use, assembly or load testing), which comprise distinct Workflows (such as modelling, analysis or simulation), which in turn are built by combining multiple Components provided by the SQUIRREL plugin. This hierarchical approach enables a modular implementation of the design process, improving its flexibility and enabling greater user control.

**Figure 4.**
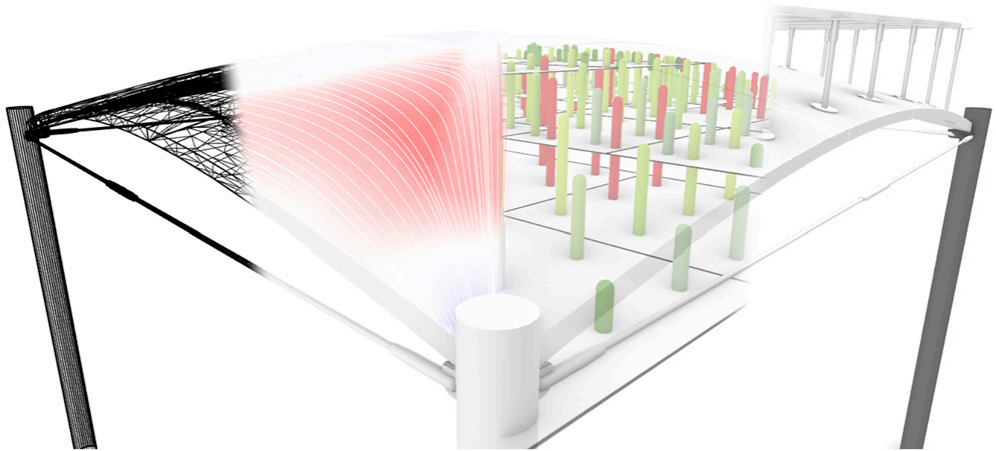
The SQUIRREL plugin aims to streamline multiple stages of designing segmented shells: (from left to right) modelling, analysis, fabrication simulation, and visualisation.

Components are combined into design Workflows, corresponding to different stages in the design process of a segmented shell (Figure 4), and grouped into toolbar tabs accordingly (Figure 2):

• Shape Modelling, in which the geometry of the shell is determined. This includes formfinding, segmentation layout and shell thickness;
• Structure Modelling, in which a structural model is built from the geometric model, by adding information about boundary conditions;
• Structural Analysis, to which the structural model is subjected to inform the design process;
• Fabrication Simulation, which enables verification that fabrication constraints are satisfied;
• Visualisation, referring to the floor itself, as well as to the design space.

**Figure 5.**
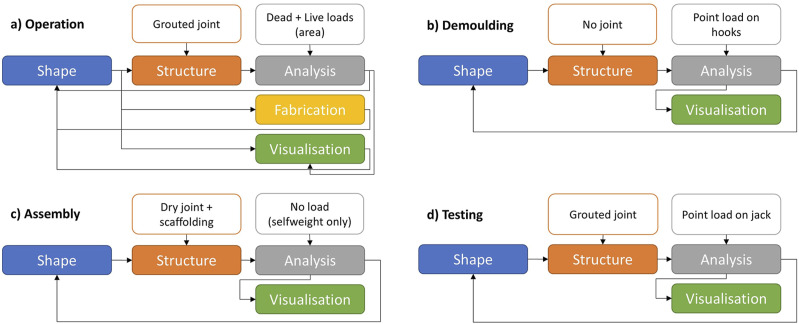
Diverse SQUIRREL workflows.

Each type of workflow generates a specific type of model, which can then be used as an input for a subsequent workflow. For example, a geometrical model, resulting from formfinding and segmentation tasks within a Shape Modelling workflow, can be passed onto a Structural Modelling workflow for determining structural fitness. However, designing a segmented shell is not necessarily a fixed process. In fact, the framework’s parametric nature provided by GH grants the design process the flexibility to accommodate different use scenarios. Also, despite GH’s acyclical dataflow, results from stages occurring downstream in the design process (Analysis, Fabrication, Visualisation) can inform design decisions in the Shape stage upstream (Figure 5).

Consequently, different Scenarios require different combinations of SQUIRREL workflows. For example, the design of the OAK prototype considers operational use, which corresponds to a particular load case. However, SQUIRREL was also used to simulate additional scenarios related to different stages in the construction of OAK, such as demoulding, assembly and testing. The difference among these scenarios can consist of different load cases, as well as a variation in the structural modelling (Figure 5). For example, the structural behaviour of the interfaces between segments during shell assembly is different from their behaviour during operation. Testing SQUIRREL in different scenarios provided an opportunity to explore the variability of the plugin’s behaviour, thus improve its development.

### Structural modelling and analysis

Digital structural models are assembled from specific inputs that include structural shell elements, supports, cross sections, materials, and loads. In the SQUIRREL design workflow, the geometric model informs the definition of some of those inputs, such as the mesh corresponding to the shape of the shell segments, shell thickness, the location of supports, and the geometry of the interfaces between segments. Other aspects of the structural model are independent of the geometry, such as material properties and the behaviour of the joints between segments. The resulting structural model can then be subjected to structural analysis.

Structural behaviour is assessed via a linear elastic analysis, using Karamba’s “AnalyzeThII” component, which implements a second-order linear elastic model through an iterative procedure with repeated updates of second order normal forces acting on the shell.^27^ This approach considers the effect of axial forces on stiffness, which therefore enables the buckling resistance to be calculated. The use of such a simple model enables design exploration, formfinding and optimisation by significantly reducing computation time compared to a more refined non-linear analysis. However, this means that geometrical and material non-linearities are not considered, even though ignoring them leads to an overestimation of buckling resistance. Therefore, further development of the tool should take them into account. For example, such overestimation can be quantified by comparing analysis results from the current SQUIRREL implementation with the ones from other FEA software such as ANSYS, or with results from physical experiments with built prototypes.

Structural performance has been the main consideration when designing the segmented shell, since it informs not only the shell’s integrity but also its efficiency in terms of concrete volume and fabrication processes. Therefore, its design is subjected to FEA to simulate and quantify its structural behaviour against three criteria: strength, informed by principal stresses; stability, informed by buckling load factors; and serviceability, informed by deflection.^16^

### Design Space Visualisation

The main objective of SQUIRREL is to find suitable design solutions for segmented shells. While optimisation processes provide a single optimal solution, or a subset of optimal solutions in the case of multi-objective optimisation processes, they do not necessarily provide information about the problem design space. In a design context, the development of tools and models benefits from the ability to visualise such a space, enabling a wider perception of the problem being solved. This premise led to the development of Design Space Visualisation (DSV), whose functionalities extend, but also depend on, Design Space Exploration (DSE).

**Figure 6.**
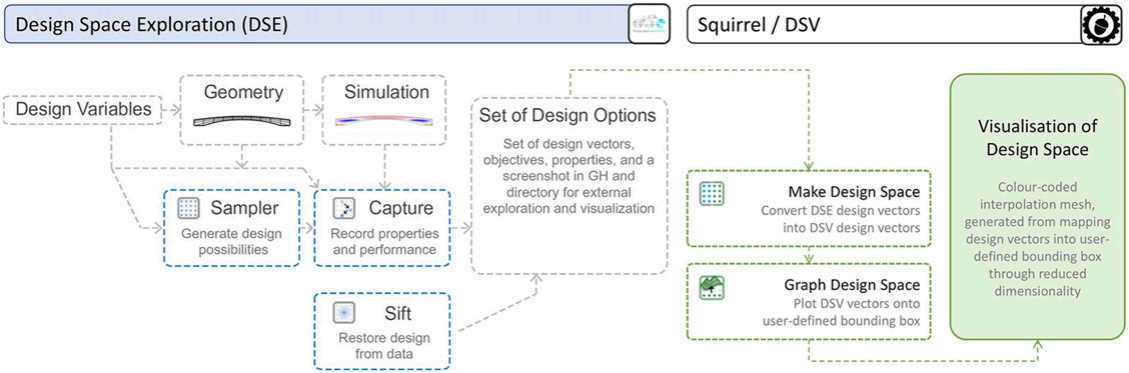
Interface between DSV and DSE, namely its implementation of the Design Catalog workflow enabled by data-driven DSE components Sampler, Capture, and Sift [adapted from^20^].

SQUIRREL uses two original components of DSE, namely Sampler and Capture. These components form a Design Catalog workflow (Figure 6), which automatically generates multiple designs in sequence, simulates their geometry,^20^ and captures the results of the structural analysis into a data structure specific to DSE, comprising multi-dimensional vector spaces composed of Design and Objective parameter values. The main intent in adopting the same data structure as DSE is to eventually make the functionalities developed for DSV available to DSE users.

Subsequently, DSV uses components developed for SQUIRREL to map the generated vectors composed of Design and Objective parameter values onto corresponding points in either 2D or 3D space, which are then used to generate a mesh, enabling the interpolation of captured values. In both cases, values from two Design parameters are mapped onto the horizontal *X* and *Y* axes. DSV 2D graphs can present values of a single Objective parameter through colour coding. On the other hand, 3D graphs can present two Objective parameters, mapping the first one onto the *Z* axis, and a second one through colour coding.

Since both the Design space and the Objective space for segmented shells are composed of more than two parameters, DSV mapping into 2D and 3D graphs implies a dimensionality reduction,^28^ which is controlled by the end-user by selecting which variables are mapped.

## Exploring shell shapes

The first design task in shaping the segmented shell is formfinding. The shell’s three-dimensional shape is determined through a formfinding process that ensures the shell works mainly in compression under the dominant load case, resulting in a membrane structure. Such a process is also constrained by the shell’s architectural requirements, namely bay geometry and maximum slab depth, thus corresponding to the main parameters of the SQUIRREL Workflow.

**Figure 7.**
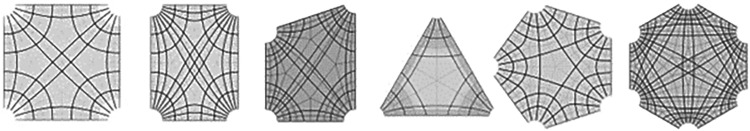
Possible bay geometries. Lines correspond to an early study on force flows within the shell, used in the design of segmentation layouts.

Therefore, the first step in the Geometry Workflow is generating a planar representation of the floor slab, executed by the SQUIRREL “Shell Plan” component. The geometry of floor slabs is defined by the building’s column layout. In construction, column layouts are typically orthogonal grids, resulting in square- or rectangular-shaped bays. Nevertheless, there might be cases where irregular quads and triangles are needed, particularly when building in an existing context Therefore, a range of possible bay shapes was included, from rectangles to polygons. Therefore, the main input of the “Shell Plan” component is a planar convex polygon, rather than rectangle dimensions (Figure 7).

Additionally, the resulting shell plan features chamfered corners. Since segmented shells are supported by columns positioned in each of their corners, such corners are chamfered to allow for load distribution in the shell-column interface.

### Formfinding

The planar shape generated by “Shell Plan” serves as the input to a formfinding process to determine the three-dimensional shape of the segmented shell. Formfinding is often used in structural design to find efficient shapes in equilibrium, being particularly useful in exploring the design of shells in compression under a dominant load case.^29^ In this approach, a membrane structure is used to find a funicular shape.^24^

SQUIRREL’s structural formfinding process, encapsulated in the corresponding “Formfinding” component, is supported by the Kiwi plugin, which uses NURBS surfaces. Given that formfinding occurs at the start of the design process, converting the shape to a polygonal mesh this early-on would greatly limit the range of geometric operations available further downstream. The Updated Reference Strategy^30^ is the formfinding method implemented in Kiwi’s Formfinding component,^29^ and therefore the one used by SQUIRREL.

**Figure 8.**
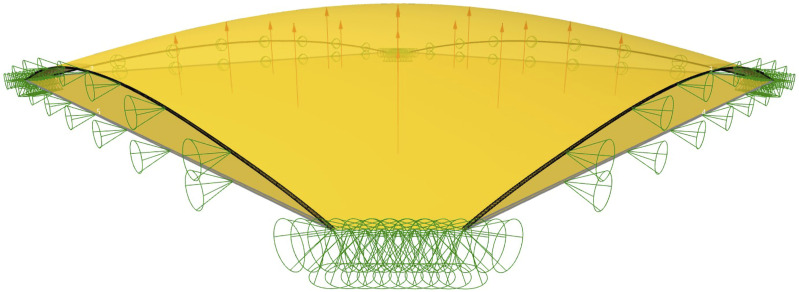
Kiwi structural model for formfinding: original flat surface and resulting surface in yellow. Supports and their corresponding restrained directions in green cones. Edges cables in grey (before formfinding) and black (after formfinding). Loads pointing upward model a reverse hanging membrane.

The “Formfinding” component automates a number of tasks required by Kiwi: first, it assembles a structural model containing information about elements, supports and loads (pink elements in Figure 8). The input chamfered shell plan is converted into a planar NURBS surface and used as a single membrane structural element. The boundary curves that connect the chamfered corners are converted into virtual structural cables, enabling a more precise control of the shell’s shape at the edges. The specific parameter values needed to define these elements are encapsulated inside the component and stated in Table 1 for reproducibility purposes, most of those values being default values from Kiwi components. Note that these parameter values do not correspond to real physical material properties, but simply serve to provide control of the shell shape through the ratio between surface and boundary values. Refinement factor values in the last row in the table define the polynomial degree of the NURBS surface and curve respectively and were adjusted above the default value of five to reduce artefacts in the form found shape.

**Table 1.** Parameter values used in the Kiwi model.

	Membrane surface	Edge cables
Young’s modulus (E)*	1 GPa	210 GPa
Density (D)*	20 kN/m^3^	78.5 kN/m^3^
Strength (fy)*	0 MPa	235 MPa
Dimension *	1 mm (thickness)	10 mm (diameter)
Refinement factor	15	8

*default values from kiwi components.

The Kiwi model includes two sets of support points, represented by cones in Figure 8. A first set of pinned supports are positioned along the chamfered corner lines. At these pinned supports, translation is restrained in all three directions, but rotation is free about all directions. A second set of supports are placed along the boundary curves corresponding to cable elements. In these support points, the only restrained degree of freedom is translation in the horizontal direction perpendicular to the edge, preventing the membrane pulling inwards due to membrane prestress, and thus ensuring that the shell’s sides are restrained in a vertical plane. The purpose of such lateral restriction is to restrict the shell’s edges to remain straight on-plan, to ensure the shell’s compatibility when applied in a structural grid. While such restriction results in an increase in stresses on the shell close to its boundaries, these are negligeable when compared to stress concentrations in the shell’s corners, according to the various structural analyses performed during the project.^16^

Finally, the Kiwi model also includes a uniformly distributed load applied in the upward vertical direction, represented by arrows in Figure 8. The assembled model is run through Kiwi’s IGA Solver, which outputs a deformed model, from which the deformed NURBS surface can be extracted. Finally, the deformed shell is adjusted to match a given target height to limit the overall depth of the floor, defined as an input of the “Formfinding” component. The adjustment factor corresponds to the ratio between the target height and the height of the deformed NURBS surface. This method uses Kiwi’s “Deformed Model” component, applying the adjustment factor to the Scale Deflections input in a single iteration.

### Shape parameters

**Figure 9.**
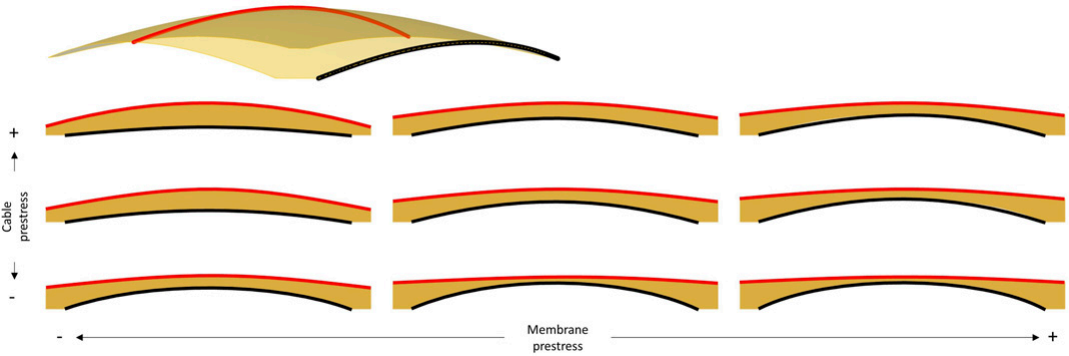
Shape exploration resulting from varying membrane and cable prestress values in formfinding process. Resulting curves in black (edge) and red (ridge).

Besides the shell plan and target depth, two other parameters grant the user control over the shell’s shape: membrane prestress (S_M_) and cable prestress (S_C_). Membrane prestress influences the overall shape of the shell surface, whereas cable prestress allows for independent control of the surface’s edges (Figure 9). Manipulating these parameters enables control of the shell’s shape while maintaining the desired compression-dominant behaviour through formfinding.

The shape definition for the OAK shell illustrates the use of Design Space Visualisation (DSV) for understanding the behaviour of the shape parameters used in formfinding. After having defined the shell’s main dimensions, a parametric study was conducted in which a set of shell shapes was form-found by varying S_M_ between 0.0 and 1.0 kN/m, and S_C_ between 0.0 and 1.0 kN. The step size was 0.125 units for both parameters. Each resulting shape was analysed using FEA for the main Objective parameters previously presented, namely principal stress and buckling load factors. This was based on the assumption that solutions with better structural performance result in thinner shells, meaning less concrete and therefore less embodied carbon. DSV was then used to visualise the results in 2D and 3D graphs.

**Figure 10.**
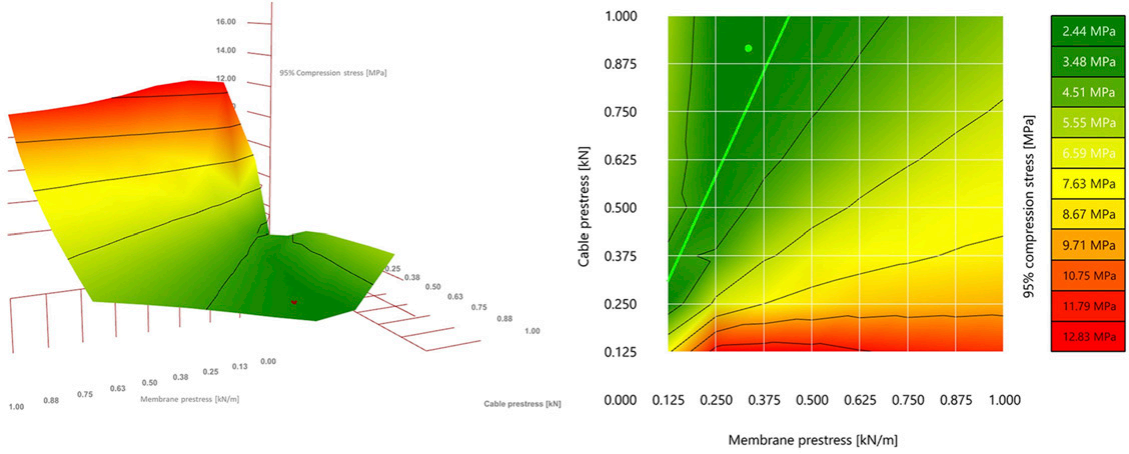
DSV-generated 3D and 2D graphs, communicating maximum compression stress values against shape parameters used for generating shape of respective shells.

A valley line is identified in the 3D graph, shown as the light green line in the 2D model (right hand side of Figure 10), whose equation corresponds to S_C_ = 1.57 * S_M_ - 0.32. Therefore, values of S_M_ and S_C_ that satisfy the equation generate solutions with a low compression stress. Also, the fact that the identified valley is not a hard edge suggests that solutions with (S_M_,S_C_) coordinates close to the line will feature a similarly low compression. This geometric approach can be useful in the search for a good solution, rather than an optimum one, which becomes more challenging in multi-objective optimisation problems. Taking the geometric analysis further, we can also note that the valley becomes wider closer to the top end along the identified line, which suggests a higher probability of finding a good solution if searching in the area of the design space that is closer to that end.

In the specific case of the OAK shell, the values for the formfinding parameters were fine tuned to S_M_ 0.335 kN/m and S_C_ = 0.915 kN (green dot in the 2D image of Figure 10). These specific values were defined through a design exploration exercise to integrate a 300 mm diameter service duct within the shell’s structural depth.^16^ For such integration, a manual exploration of the design space was conducted to find a shell shape that would not intersect the duct. Identifying a specific ratio of S_M_/S_C_ from the DSV plot significantly narrowed down the fine-tuning process, rather than having to explore random combinations of S_M_ and S_C_.

## Exploring segmentation layouts

**Figure 11.**
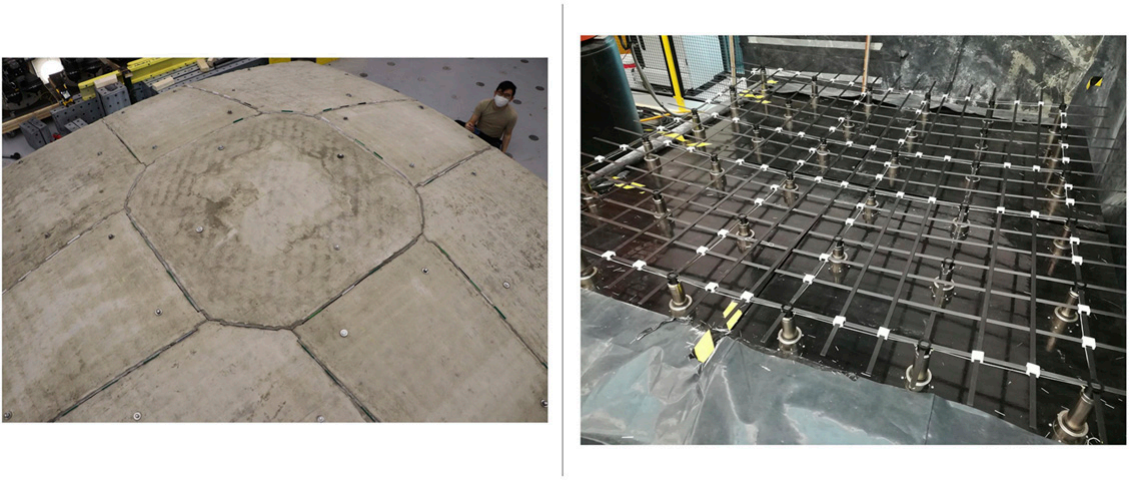
Prefabricated segments assembled into a whole shell (left); Reconfigurable mould actuated by a set of vertical mechanically driven pins and connecting flexible formwork (right). Photo credits: ©Robin Oval.

The geometry resulting from the formfinding process corresponds to the overall shape of segmented shells. The proposed shells are to be produced off-site, to benefit from the precision and controlled environment of a manufacturing plant, to leverage automation and minimise waste, reducing the environmental impact by limiting construction waste in casting and formwork. However, due to manufacturing, transportation and assembly constraints, the shell cannot be built as one single element, but rather must be segmented into multiple, smaller elements (Figure 11, left).

### Segment manufacturing

The segmented shells are manufactured thanks to an innovative reconfigurable mould system to create the shell’s casting surface and a robotic concrete spraying system to fabricate the shell with little waste. The reconfigurable mould system actuates a flexible formwork on which sits a custom timber frame for shaping the concrete shell. It consists of a set of square modules consisting of a grid of actuated pins, which can be laid out into a larger mould, used to fabricate shell segments (Figure 11, right).^15^ The dimensions of the modules and its components are set as variable parameters in SQUIRREL, accommodating for changes to the flexible mould specifications.

Enabled by the compression-dominant behaviour of the segmented shells, the joints between the segments are dry, with no mortar or grout, facilitating their future disassembly. Half-joint shear keys along the interfaces (Figure 11, right) prevent out-of-plane sliding failure in the direction where the compression forces in the shell induce shear at the interfaces.^15^

The shell’s structural behaviour and its fabrication process drive the design of the segmentation layout, which follows a set of geometrical constraints. Firstly, segmentation should be as orthogonal as possible to the flow of compressive forces, to prevent in-plane sliding failure.^8^ Most importantly, the segments must fit within the boundaries of the reconfigurable mould so they can be manufactured.

**Figure 12.**
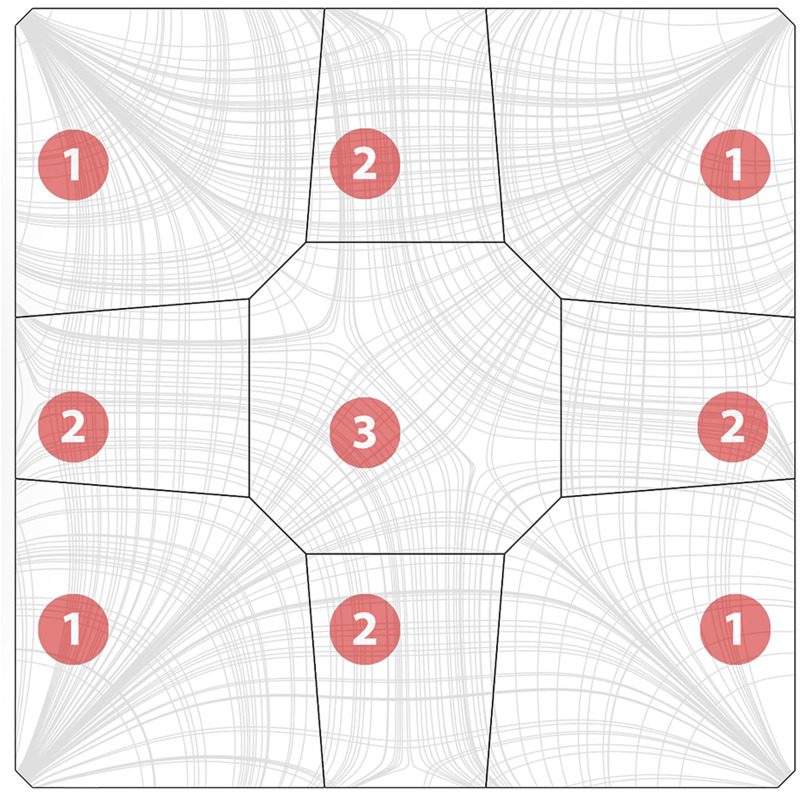
Segmentation layout for the OAK shell, compared with its principal stress lines. (Legend: 1- Corner segment; two- edge segment; 3- key segment).

Figure 12 illustrates the flow of compressive forces, as well as the resulting segmentation layout used in the OAK prototype, which features an octagonal central segment and trapezoidal edge segments, so that the dominant compression forces prevent the edge segments from sliding out.^16^ This layout resulted from a preliminary design exploration of the OAK shell, in which various layouts were explored to understand the minimum number of segments for a particular span. Apart from the number of segments, this study also considered other parameters, including number of different segments, number of different module layouts, the existence of a central key segment, and aesthetics.

While this preliminary design study featured some degree of automation, namely through automated checks of structural integrity and performance, the design exploration was performed manually. Nevertheless, it informed the constraints in designing segmentation layouts, which in turn informed a subsequent experiment with automating such design in the scope of SQUIRREL.

### Automatic generation

**Figure 13.**
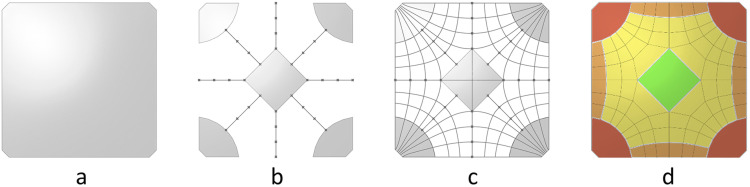
Shell segmentation sequence: (a) initial shell surface, (b) source points placed in the surface space between corner segments and key segment; (c) resulting stress lines; (d) resulting segmentation layout including corner segments (red), edge segments (orange), interior segments (yellow) and key segment (green).

A SQUIRREL component was developed to automate the generation of the segmentation layout, considering the aforementioned set of geometrical constraints. The resulting shell segments are named according to their position within the shell. Therefore, four “corner” segments connect the shell to the supporting columns, and the “key” segment is located at the shell’s apex at its centre. The remaining segments can be further differentiated into “edge” segments, positioned along the shell’s edge, and the remaining “interior” segments (yellow in Figure 13(d)).

The automated process begins by generating a network of principal stress curves from a form-found surface representing the shell, using SQUIRREL’s “Stress Lines” component (Figure 13(c)). This component uses Karamba’s API to implement its “Line Results on Shells” component in the “PrincStress” option, to generate sets of Principal Stress Lines on Shells. Karamba separates such lines into two sets, corresponding to curves tangent to first and second principal stress directions. In the case of the generated shells, which are predominantly under compression, the highest absolute stresses are along the second principal direction, which directly flow to the support, as opposed to the transverse ones, which circle around the supports. Therefore, first principal direction curves will be referred to as “circumferential stress lines”, and second principal direction curves will be referred to as “radial stress lines” (Figure 13(c)). Even though these lines are not circumferential per se, using the terms “circumferential” and “radial” instead of “first” and “second” facilitates visually identifying the two opposite sets in the curve network for the purpose of segmentation.

Karamba calculates stress lines by tracing paths from specified start-points on the shell surface. To generate a meaningful segmentation layout, starting points are placed on geodesic curves that connect the midpoint of each boundary curve to the shell’s apex (Figure 13(b)). Points on geodesics that start in the shell corners are used to calculate circumferential stress lines, whereas those on geodesics that start at the mid-edges are used to calculate radial stress lines, which converge at the shell’s supports.

The “Segmentation Layout” component uses the resulting curve network to generate a layout by simplifying each curve into a polyline containing the intersection points of the original curve with all the curves in the opposite set. The resulting layout corresponds to the set of regions that result from vertically projecting all polylines onto the surface. Consequently, the boundary of each shell segment has a polygonal horizontal projection, which facilitates the fabrication process by allowing linear boundary frames.

Users can specify the dimensions of the different types of segments by passing them as user inputs into the automated segmentation process. Input segment dimensions translate into distances between points on the geodesics, which in turn determine the distance between stress lines in the curve network and consequently the size of shell segments.

### Fabrication simulation

**Figure 14.**
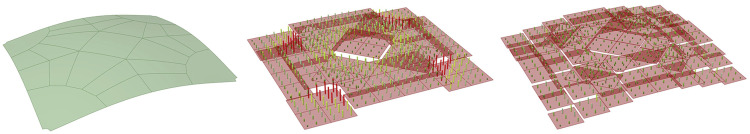
Left: surface set corresponding to a segmented shell; Centre: simulation of reconfigurable mould with constant height. Right: simulation of reconfigurable mould in which modules are lifted individually.

A SQUIRREL component capable of simulating the reconfigurable mould was developed to validate segmentation layout, consequently determining the required pin heights for a given segment and ensuring that the height differential along the segmented extrados did not exceed the maximum pin travel-height of the mould. If any of the simulated pin heights is larger than the maximum allowable pin height, three strategies were considered for implementing the simulation component. The first strategy consists of lifting each of the mould’s modules at different heights relative to each other. Figure 14 shows the simulated reconfigurable mould for the segmented shell surface on the left, with constant and differentiated module heights, where pins that exceed maximum height are highlighted in red.

**Figure 15.**
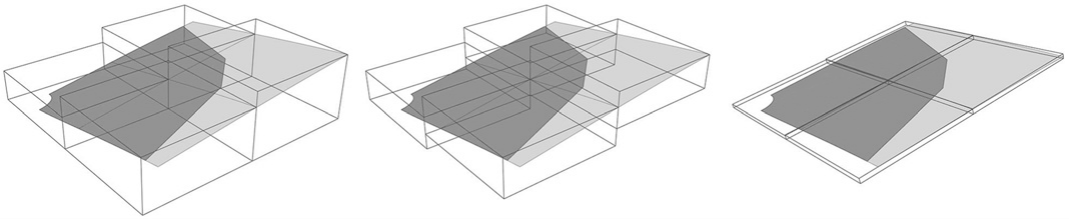
Left: Default bounding box; centre: lifted bounding box; right: oriented bounding box (representation: Darker surface = segment; lighter surface = plane best fit to segment surface; dark wireframe = bounding box).

**Figure 16.**
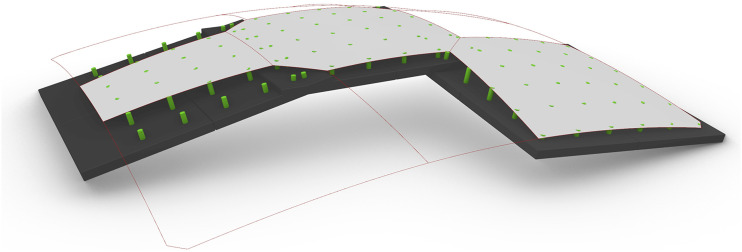
Simulation of the reconfigurable mould for three unique shell segment designs (reconfigurable pins in green, shell bottom surface in grey, base of mould in black).

The second strategy consists of rotating the segment’s orientation in three dimensions relative to the module’s top plane, based on their minimum volume bounding box.^31^ Such strategy can considerably reduce the needed pin height, especially when compared with the default and lifted modules (Figure 15), since the required pin height would depend only on the segment’s curvature, but not on its position within the whole shell. Figure 16 illustrates the potential application of the rotated bounding box to fabricating the segments of the OAK prototype. While the reconfigurable mould did not allow for reorientation nor differentiated module heights, subsequent designs of the equipment might accommodate such features.

### Structural modelling of shell segments

Segmentation tasks, automated or not, result in a Boundary Representation (Brep) of the segmented shell, consisting of a set of joined trimmed surfaces corresponding to the medial surface of each segment. On the other hand, models in Karamba are represented by a set of shell elements, each defined by a triangular mesh. Therefore, medial surfaces in each segment are converted into meshes using Karamba’s ‘MeshBreps’ component, with the component’s parameters relative to resolution being set to Target mesh size MRes = 0.04 m, and Edge refinement factor ERef = 0.66, While resulting in a fairly high resolution mesh, such parameter values allow for an increase in mesh vertices at the shell’s corners, where it interfaces with the column, to avoid stress concentrations. To avoid the orthogonal grid meshes, which might bias FEA in orthogonal directions, the resulting meshes were smoothed by setting the component’s parameters Smoothing step size SStep = 1 and Smoothing iterations SIter = 3.

**Figure 17.**
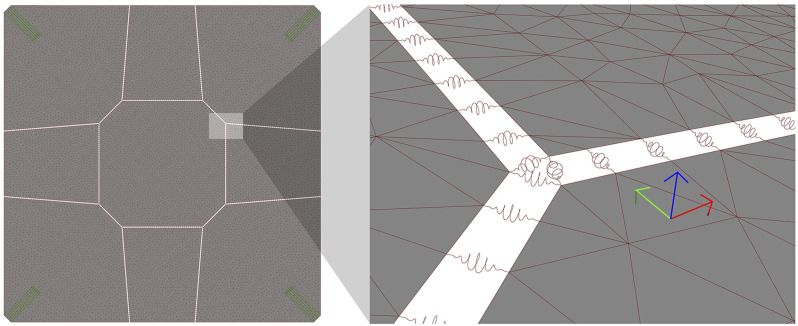
Left: oriented supports (green) in the Karamba structural model; Right: detail of segment interfaces (gap size exaggerated for illustration purposes), with local coordinate systems per spring (*X* direction in red, *Y* direction in green, *Z* direction in blue).

The structural model also comprises four sets of support points, one set for each corner of the segmented shell. These supports are oriented along the direction of the corner edge, with unconstrained rotation about all axes. Translation is fixed in the vertical *Z* axis, corresponding to the column’s reaction force countering gravity, and in the direction perpendicular to the corner edge towards the inside of the shell, corresponding to the reaction force countering the shell’s horizontal thrust (Figure 17, left) but unrestrained along the corner edge.

A reliable structural model of the segmented shell needs to address its behaviour at the interfaces between segments. The location of such interfaces is extracted from the topological information of the segmented shell’s Brep, each interface corresponding to a Brep’s internal edge, that is, an edge that shares two surfaces.

In the structural model, the interface between any pair of segments is modelled as a set of springs distributed along their shared edge, connecting neighbouring vertices between corresponding segments’ meshes. The number of spring locations for each internal edge is dependent on the parameters used in the surface to mesh conversion using ‘MeshBreps’, given that the springs need to coincide with mesh vertices for a correct structural model. In fact, one of Karamba’s ‘MeshBreps’ component inputs, IPts, receives a list of points to be included in the meshes it generates. Therefore, we can force matching vertices between segments by previously generating a set of points distributed along the segments boundaries and passing it into the meshing component’s IPts parameter.

In Karamba, springs are implemented as beam elements characterised by a cross section of the type ‘Spring’. Despite being allowed in Karamba, zero-length beams cannot be used to geometrically define the orientation of the beam, which is needed when defining stiffness values for different directions. Therefore, to facilitate the correct orientation of the spring elements, a small gap (2 mm) was added by offsetting all the segments at the internal edges (in Figure 17, right – the gap size is exaggerated for illustration purposes). Each spring could then be defined by the points in the segments’ offset edges that were closest to the corresponding spring location. A sensitivity study was conducted to confirm that the added gap does not influence the analysis results. The introduction of a gap for defining the springs dictates that the offset segments surfaces are used downstream in the workflow rather than the original shell segments, namely for subsequent conversion into polygonal meshes.

### Spring stiffness parameters

Apart from its geometry, a ‘spring’-type cross-section is characterised by translational and rotational stiffness parameters in its three local directions. To determine such parameters, a simplified model of linear elastic material behaviour was assumed, which does not consider non-linearities resulting from the fact that the joints have stiffness in compression but not in tension, and that the shear keys block restrain translation in one direction, resulting in partial interlocking. In the context of this simplified model, values for translational stiffness were determined from material properties, although not considering the effects of friction. However, quantifying rotational stiffness would require more advanced shell theory calculations, considering not only material properties but also the geometry of the shear keys at the joints.

Therefore, to determine a reasonable value for rotational stiffness (C_R_) model, a parametric study was conducted using SQUIRREL, in which the structural performance of an unsegmented shell was compared against that of a segmented shell, implemented as explained in the previous section. The parametric study tested the value of *n* in the calculation C_R_ = 10^*n*^ [kNm/rad], in which *n* varied between −2 and 4, since prior analysis showed negligeable variation for values beyond this interval. To assess the consistency of values, variations of load values and asymmetric load patterns were included in the design space.

**Figure 18.**
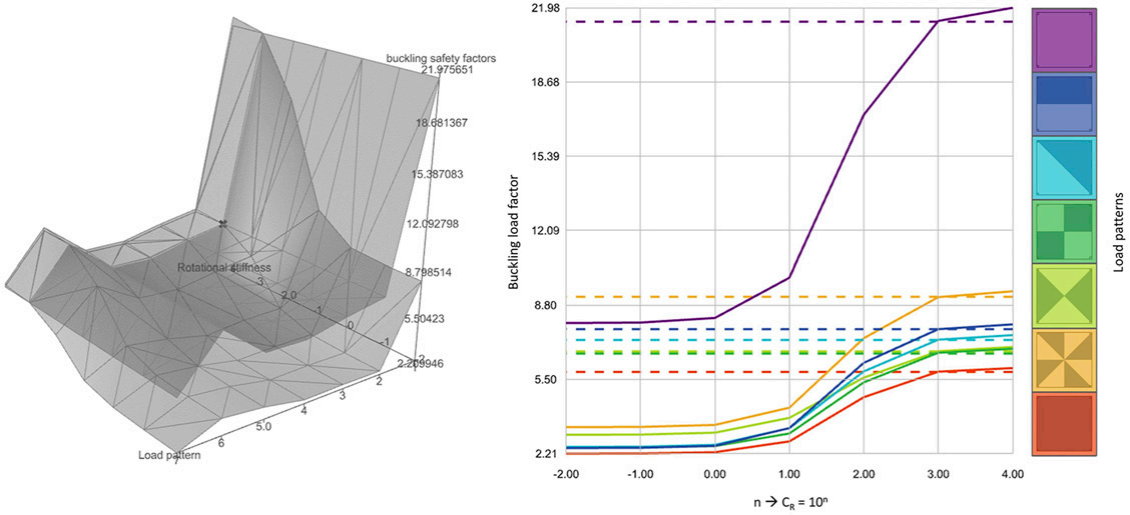
DSV-generated 3D and 2D graphs, communicating variation of buckling load factors in a segmented shell (continuous lines) with varying rotational stiffness and asymmetric load patterns in comparison with an equivalent unsegmented shell (dashed lines) (load patterns adapted from^32^).

DSV was used to visualise the results of the parametric study and better understand the shell’s structural behaviour as a function of C_R_. Figure 18 shows the logarithmic variation of C_R_ for different load patterns and resulting buckling load factors (BLF), showing the behaviour of both segmented and unsegmented intersecting at *n* = 3, resulting in C_R_ = 10^3^ kNm/rad. While variation is more visible for BLF, other structural performance indicators such as principal stress and deflection were also calculated, and in all of them variation was consistent in the intersection in *n* = 3.

Consequently, when designing the OAK prototype, inter-segment springs featured high translational stiffness (C_t_ = 10^7^ kN/m) in all three directions, which is consistent with the axial stiffness of concrete under compression of E.t [kN/m] for a shell, with E = 30 GPa and t = 50 mm, resulting in E.t=(1.5)10^6^ kN/m. On the other hand, rotational stiffness around the *Y* direction along the segment edge is very low (C_r_ = 10^−2^ kNm/rad), whereas rotational stiffness around the remaining two directions relatively high (C_r_ = 10^3^ kNm/rad),^16^ as suggested by the parametric sensitivity analysis, enabled by SQUIRREL. While these values were used for designing the OAK shell, such values should be calibrated against additional physical test results of built shells.

The original purpose of this study was to predict the behaviour of the OAK prototype, through a comparison between segmented and unsegmented shells, which resulted in adopting a very low rotational stiffness value along the joint. However, the study can also be useful to inform further design of the joints. The study suggests that that providing the joints with rotational stiffness of 10^3^ kNm/rad would almost triple the BLF for the segmented shell, leading to potential material reduction, albeit at the expense of more complex joints.

## Conclusion

Throughout the presentation of the SQUIRREL plugin in this paper, we can identify situations reflecting different balances between full automation and user interaction, which illustrate the argument that full automation is not always needed, or even useful. Since we are addressing SQUIRREL’s development phase, the term “user” in this context corresponds to the research team in charge of development.

As mentioned previously, there has been an effort to fully automate some of the more repetitive tasks. However, full automation is not always a suitable approach, as demonstrated by the design exploration employed in setting the shape parameter for the formfinding process, explained in Section 3.2. In this case, DSV was used to understand the interdependency between membrane and cable prestress, which helped identify a region along which a good solution could be found. A fully automated optimisation algorithm would likely identify a single optimum point, whereas the DSV provided a more informed search space, which could be used for a more flexible design exploration process towards building integration, and particularly relevant when dealing with multiple conflicting design objectives.

A similar example can be found in defining the spring stiffness parameters for modelling the interfaces between shell segments, as explained in Section 4.4. In this case, the user can specify the stiffness value for a specific rotation direction, corresponding to the hinge action between segments. In the scope of development, the main objective of exposing such parameter was to conduct parametric studies, and later to calibrate the system according to subsequent physical tests in the project. However, in a scenario of operational use of SQUIRREL, exposing this parameter enables it to be modified for different types of connections between shell segments. For example, shear keys could be replaced with, or complemented by, demountable steel plate connectors, providing much higher stiffness at the interface, and more easily measured. Also, the tool and its analysis can be used to inform the design and detailing of the joints, through similar sensitivity studies, which are particularly important for exploring the design of complex and novel elements with higher uncertainty. An alternative fully automated approach would be to have an optimiser determine which combination of stiffness values would better correspond to an unsegmented shell. While potentially faster, such an approach would provide less useful information to the adjustment of these parameters according to physical tests.

In these situations, the use of optimisation tools might have been quicker, requiring less human supervision, or the generation of fewer solutions. However, in the scope of research, the understanding of the system’s behaviour is crucial, and therefore the additional time needed to generate the DSV graphs was considered necessary.

Such an approach also determined the nature of the SQUIRREL plugin, whose aim was to expose the design process by providing multiple components that perform different functions, as well as example files corresponding to predetermined combinations of those components. Had it been fully implemented using a black box approach, SQUIRREL would generate an optimised shell with a set of inputs, albeit enabling less flexibility for further design exploration or adoption of the framework by others.
